# Quantitative study on water and glycosaminoglycan contents based on T_2_ relaxation time of intervertebral disks

**DOI:** 10.3389/fmed.2026.1889003

**Published:** 2026-08-10

**Authors:** Bing Qin, Xiaofeng Jia, Wenxin Ni, Lu Zhou, Fan Gao, Zhiyu Qian, Qiaoqiao Zhu

**Affiliations:** 1 Department of Biomedical Engineering, Nanjing University of Aeronautics and Astronautics, Nanjing, China; 2 Nanjing Hospital of Traditional Chinese Medicine, Nanjing, China; 3 College of Information Engineering, Taizhou University, Taizhou, China

**Keywords:** glycosaminoglycan content, intervertebral disk, quantification, T_2_ mapping, water content

## Abstract

**Background:**

Water and glycosaminoglycan (GAG) are essential for intervertebral disk biomechanics and their loss marks early degeneration, yet the quantitative relationship between T_2_ relaxation time and contents of water and GAG remains unclear due to low-resolution sampling.

**Purpose:**

To establish the quantitative relationships between T_2_ relaxation time and key biochemical components of intervertebral disk (water and GAG).

**Methods:**

Five fresh bovine caudal spine segments were sectioned into 13 anatomical zones for high spatial resolution sampling and spatially-matched analysis. Multi-echo MR images were obtained on a 3T MR imaging scanner and the T_2_ relaxation time was calculated, and biochemical assay for water content (tissue volume fraction) and GAG content (%dry weight) were conducted. The quantitative relationships between T_2_ relaxation time and water content, T_2_ relaxation time and GAG content were established using linear regression and validated.

**Results:**

T_2_ relaxation time, water content, and GAG content showed a similar spatial distribution pattern within the IVD, with highest values in the central nucleus pulposus and decreasing toward the peripheral annulus fibrosus. T_2_ relaxation time correlated significantly with water content (R^2^ = 0.8, *p* < 0.05), T_2_ relaxation time correlated significantly with GAG content (R^2^ = 0.74, *p* < 0.05).

**Conclusion:**

The water content and GAG content could be predicted relatively accurately based on the T_2_ relaxation time through quantitative models.

## Introduction

The lumbar intervertebral disk (IVD) played a significant biomechanical role as the main joints of the spine, such as transmitting mechanical loadings and providing flexibility to the spine (1, 2). The biomechanical function of IVD was primarily sustained by two components: water, which was the main medium for the transmission of mechanical loading and the absorption of energy, and glycosaminoglycan (GAG), the side chains of proteoglycans (3, 4). The fixed negative charge carried by GAG generates an osmotic pressure, which drew and retained water, thereby creating the swelling pressure that maintained high water content within the IVD (5–9). The loss of GAG and the reduction of water content were signs of early IVD degeneration were reported in numerous studies (10, 11). Therefore, the quantitative, spatially-resolved assessment of these components was helpful for a better understanding of IVD biochemistry and the early detection of degenerative changes.

Magnetic Resonance Imaging (MRI) was a primary non-invasive diagnostic tool in clinical practice, and T_2_ mapping was an important method for assessing tissue water content. T_2_ mapping was used to evaluate the water content of tissues in numerous studies (12–14). Studies have reported that the T_2_ relaxation time of degenerate IVD was shorter than that of normal IVD (15, 16). Previous studies correlating T_2_ relaxation time with biochemical composition generally evaluated broad nucleus pulposus (NP) and annulus fibrosus (AF) regions (12). MRI-based water content quantification studies have similarly reported measurements at the NP and AF levels (13). High spatial resolution biochemical studies have demonstrated regional gradients in water and GAG content, but did not include spatial matching with MRI-derived T_2_ relaxation time (9). In a T_2_ mapping study, Detiger et al. noted that correlating a small biochemical specimen with an MRI value averaged over the whole NP may weaken imaging-biochemistry associations because of scale mismatch (17). To explore the quantitative relationships between MRI signal and major IVD biochemical components, thereby advancing non-invasive assessment of biochemical components based on the MRI signal, this study used a high spatial resolution sampling approach. The quantitative relationships of T_2_ relaxation time with water and GAG contents were evaluated with precise spatial matching. The linear regression models to predict water and GAG contents directly from T_2_ mapping data were used. This study is helpful for applying clinical T_2_ mapping as a non-invasive tool for quantifying the water and GAG contents within the IVD, providing new potential for clinical diagnosis and personalized biomechanical analysis of IVD degeneration.

## Materials and methods

### Specimen collection

Five fresh bovine caudal spine segments were obtained from a local slaughterhouse. The segments were sealed in plastic bags and stored at room temperature for 6 h before imaging. Subsequently, they were frozen and stored at −80 °C. For the subsequent dissection and biochemical analysis, the specimens were thawed in a water bath at 25 °C and equilibrated to room temperature.

### Measurement of T_2_ relaxation time

All scans were performed on a 3T Siemens Prisma scanner (Siemens Healthineers, Erlangen, Germany). Specimens were positioned on the scanner table with the coil aligned to the scanner’s z-axis. A localizer scan was first acquired to confirm positioning.

T_2_ relaxation time was measured using a multi-echo spin-echo sequence. This sequence samples signal decay across multiple echo times (TEs) and allows voxel-wise estimation of T_2_ relaxation time by mono-exponential fitting. A midline sagittal image was acquired using a multi-echo sequence. The sequence was configured to sample a series of echo times for T_2_ quantification by employing multiple excitations with an echo train length of six per excitation. Imaging parameters included: repetition time (TR) of 1,950 ms, six echoes acquired at TEs of 10.5, 21.0, 31.5, 42.0, 52.5, and 63.0 ms, with an inter-echo spacing of 10.5 ms,, slice thickness of 2 mm, matrix size of 512 × 512, and receiver bandwidth of 375 Hz/pixel.

The acquired multi-echo images were imported into 3D Slicer software (version 5.8.1^1^). For each voxel, T_2_ relaxation time was estimated by fitting signal intensities across the six echo times to the mono-exponential model. The T_2_ relaxation time, expressed in ms, was derived as the decay time constant (T_2_ relaxation time) from the fit. Parametric T_2_ maps were generated to visualize the spatial distribution of T_2_ relaxation time within the IVD. Before spatial matching, the acquired images and generated T_2_ maps were assessed for image quality and artifacts by an MRI engineer and a clinician. Only image sets considered suitable for spatial matching were included in the analysis.

**FIGURE 1:**
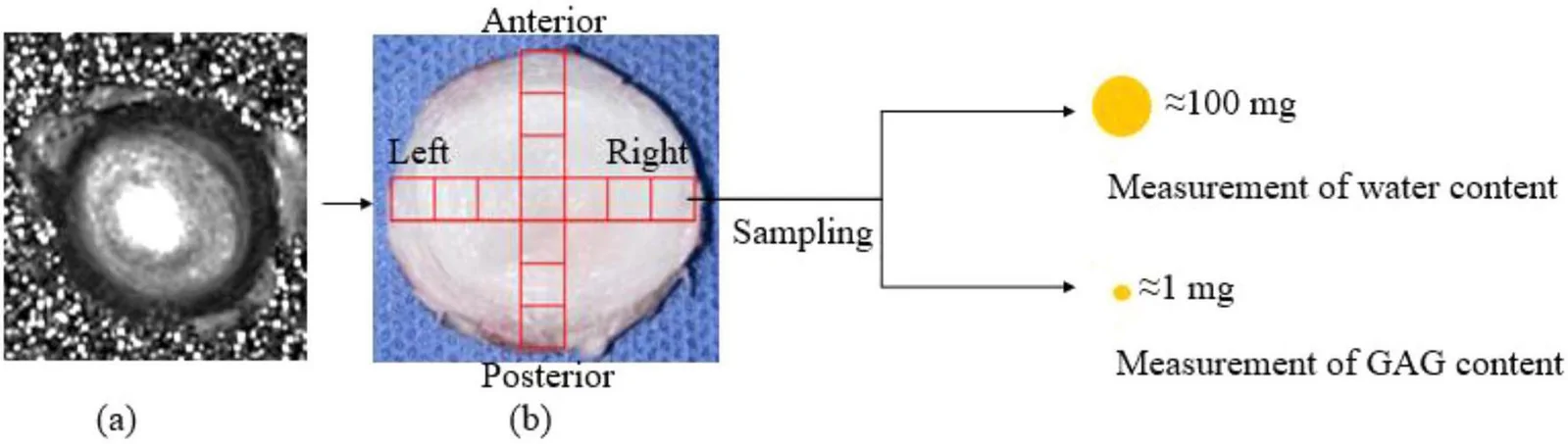
T_2_ mapping and spatial sampling design. **(a)** T_2_ relaxation time maps of the mid-transverse plane of intervertebral disk (IVD). **(b)** Schematic of the 13-zones sampling design. Seven evenly spaced sampling zones were defined from anterior to posterior in the sagittal direction and from left lateral to right lateral in the coronal direction. Because the central zone was shared, 13 sampling zones were obtained per IVD. The same sampling design was applied to the T_2_ map and tissue sampling of each IVD.

Each IVD was oriented along the sagittal and coronal directions. Seven evenly spaced sampling zones were defined from anterior to posterior in the sagittal direction and from left lateral to right lateral in the coronal direction. Because the central zone was shared, 13 sampling zones were obtained per IVD (Figure 1). The same sampling design was applied to the T_2_ map and tissue sampling of each IVD. The mean T_2_ relaxation time of all voxels within each sampling region on the T_2_ map was paired with water and GAG content measurements from tissue collected at the corresponding zone.

### Measurement of IVD water content

The spatial distribution of volumetric water content within the IVD was determined by combining volumetric measurement based on Archimedes’ principle with gravimetric analysis following lyophilization. Approximately 100 mg tissue specimens were harvested from each sampling zone, immediately weighed to obtain the wet mass, and the tissue volume was measured using Archimedes’ principle. Specimens were lyophilized (freeze-dried) using a standard protocol consisting of a 6-h precooling phase followed by a 24-h primary drying phase, and then weighed again to obtain the dry mass. The volumetric water content was calculated as the mass of water (wet mass minus dry mass, assuming a water density of 1.0 g/cm^3^) divided by the measured tissue volume.

### Measurement of IVD GAG content

The GAG content was quantified using the 1,9-dimethylmethylene blue (DMMB) dye binding assay. Approximately 1 mg of tissue was harvested from each sampling zone of the IVD. The tissue specimens were lyophilized using a standard protocol (6-h precooling followed by 24-h primary drying), and the dry mass was immediately recorded post-lyophilization. The dried samples were then processed using a commercially available kit (GAG assay Kit, GMS 19239.2, Genmed Scientifics Inc., USA). Briefly, the dried samples were digested with proteinase K at 56 °C for 16 h to ensure complete GAG extraction. The extracted GAG was subsequently stained with the DMMB reagent, followed by a dissociation step to ensure specific binding. The absorbance of the resulting solution was measured at 656 nm using a microplate reader (CMax Plus, Molecular Devices, USA). GAG content within each sample was determined by comparison with a calibration curve prepared from a GAG standard of known concentration. The GAG content was finally expressed as a percentage of dry tissue mass.

### Statistical analysis

Linear regression analysis was performed to model the relationships between T_2_ relaxation time and tissue biochemical composition (water content and GAG content). A randomly selected subset comprising half of the samples was used to establish linear regression models correlating T_2_ relaxation time with water content and GAG content, respectively. These models were then applied to predict the water content and GAG content of the remaining half of the samples. The accuracy of the predictions was assessed by comparing the predicted values with the experimentally measured ones, quantified using both the root mean square error (RMSE) and the coefficient of determination (R^2^). All statistical analyses were performed using Microsoft Excel, with statistical significance assumed at *P* ≤ 0.05.

## Results

### Spatial distribution patterns of T_2_ relaxation time, water content, and GAG content within the IVD

**FIGURE 2:**
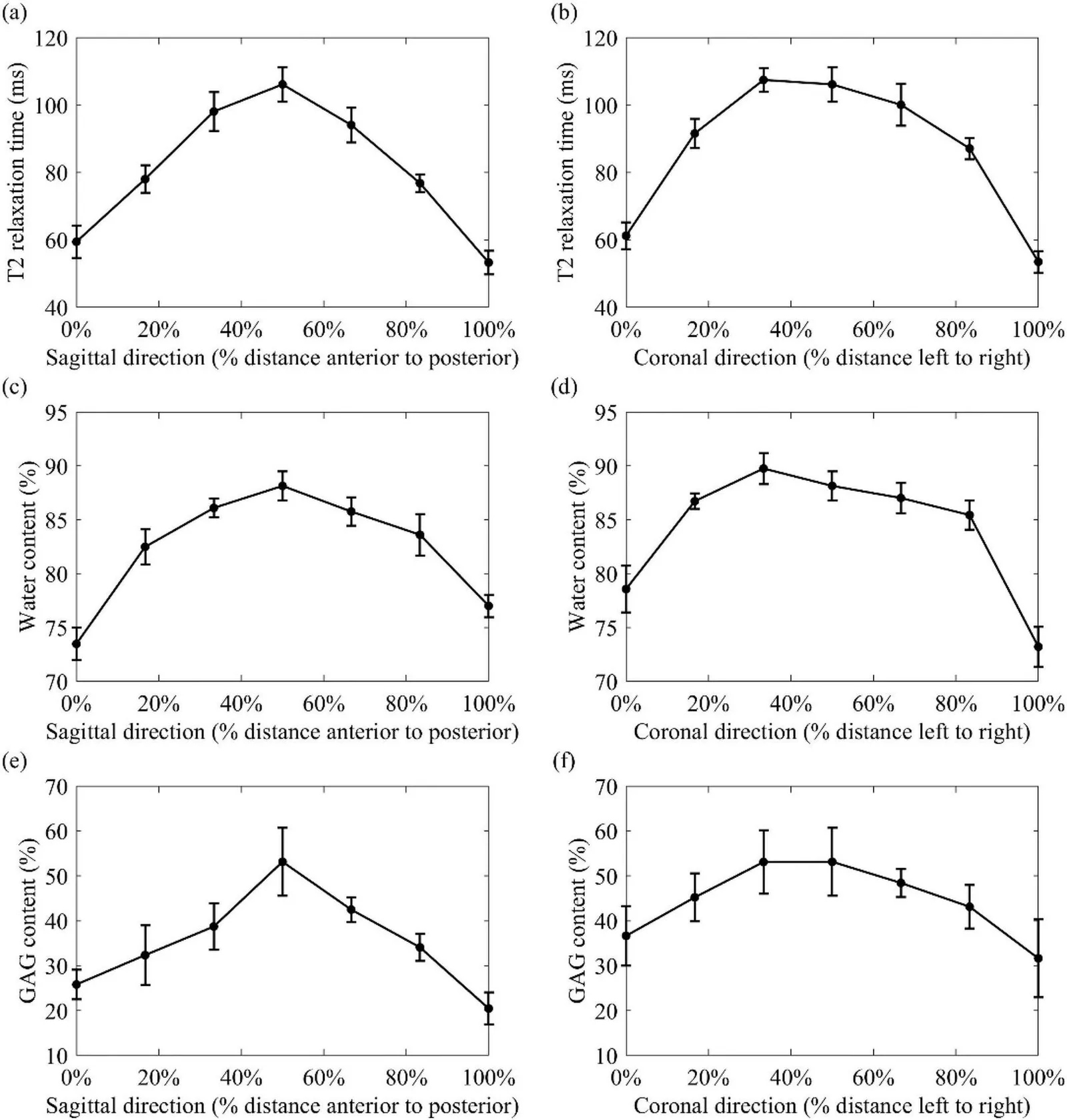
Mean spatial distributions of T_2_ relaxation time, water content, and glycosaminoglycan (GAG) content along the sagittal and coronal directions. Data were presented as mean ± SEM across five intervertebral disks (IVDs). **(a,c,e)** Showed the sagittal direction from anterior to posterior. **(b,d,f)** Showed the coronal direction from left to right. **(a,b)** Showed T_2_ relaxation time. **(c,d)** Showed water content. **(e,f)** Showed GAG content.

Quantitative analysis revealed consistent spatial gradients across the IVD for all three parameters. T_2_ relaxation time (106 ± 13 ms), water content (88% ± 3%), and GAG content (53% ± 18%) were highest in the central region and decreased progressively toward the peripheral AF. The distribution patterns of T_2_ relaxation time, water content, and GAG content were qualitatively similar throughout the IVD (Figures 2a–f).

### Correlation between T_2_ relaxation time and water content and GAG content: linear regression analysis

**FIGURE 3:**
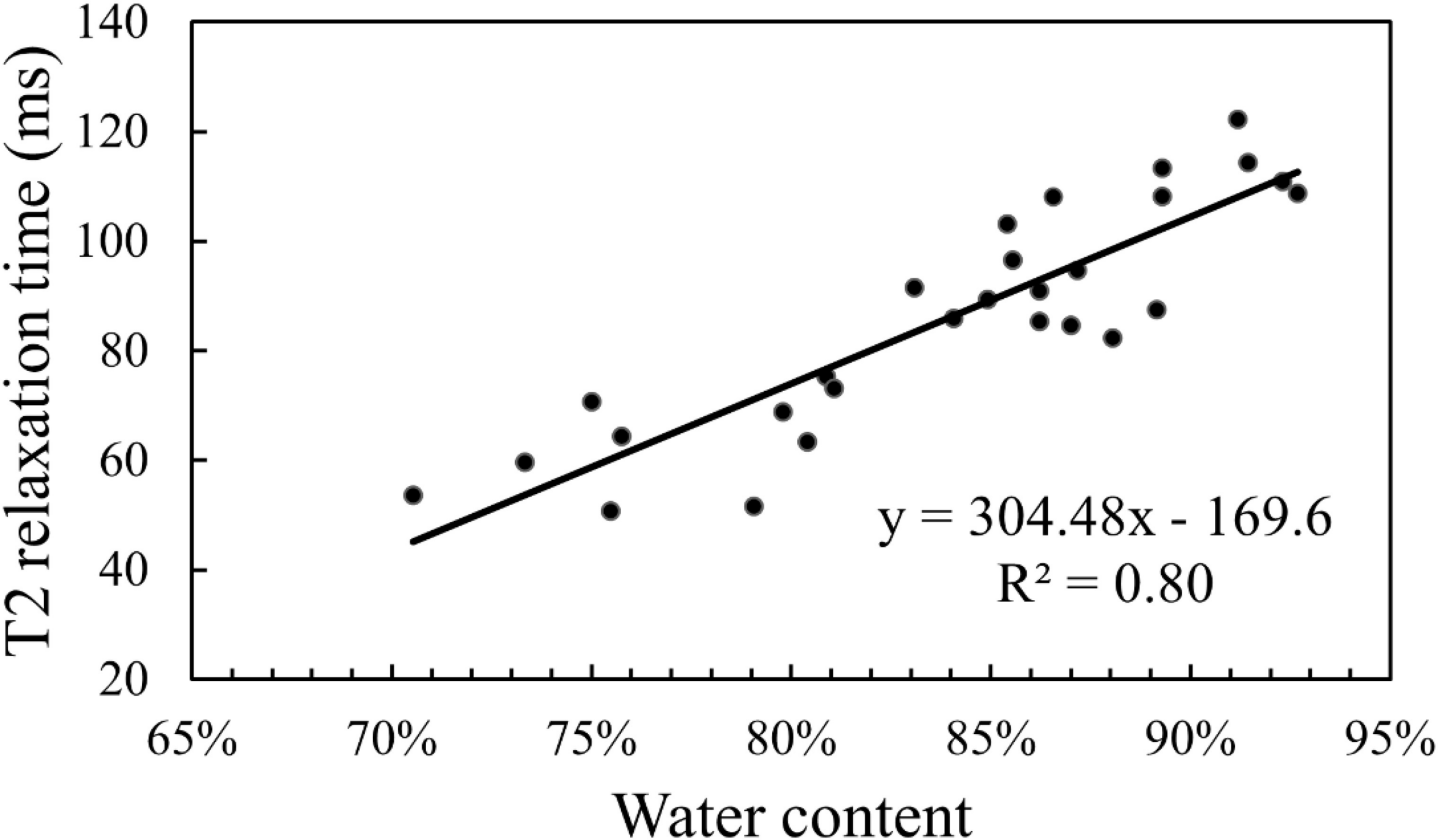
Correlation of T_2_ relaxation time with water content.

**FIGURE 4:**
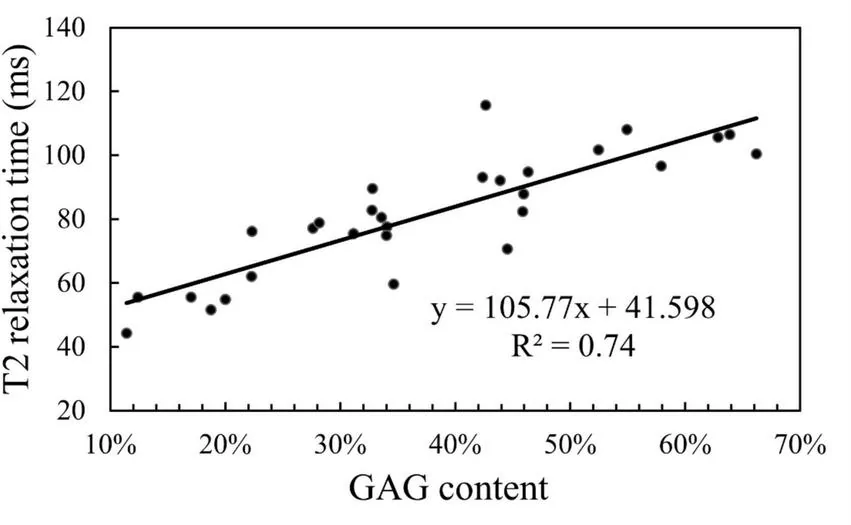
Correlation of T_2_ relaxation time with glycosaminoglycan (GAG) content.

Linear regression analysis showed a significant positive correlation between T_2_ relaxation time and water content across all samples (*P* < 0.05), indicating a strong linear relationship (R^2^ = 0.8, slope = 305) (Figure 3). Consistently, T_2_ relaxation time also showed a significant positive correlation with GAG content across all samples (*P* < 0.05), indicating a strong linear relationship (R^2^ = 0.74, slope = 106) (Figure 4).

### Validation of the quantitative relationship

**FIGURE 5:**
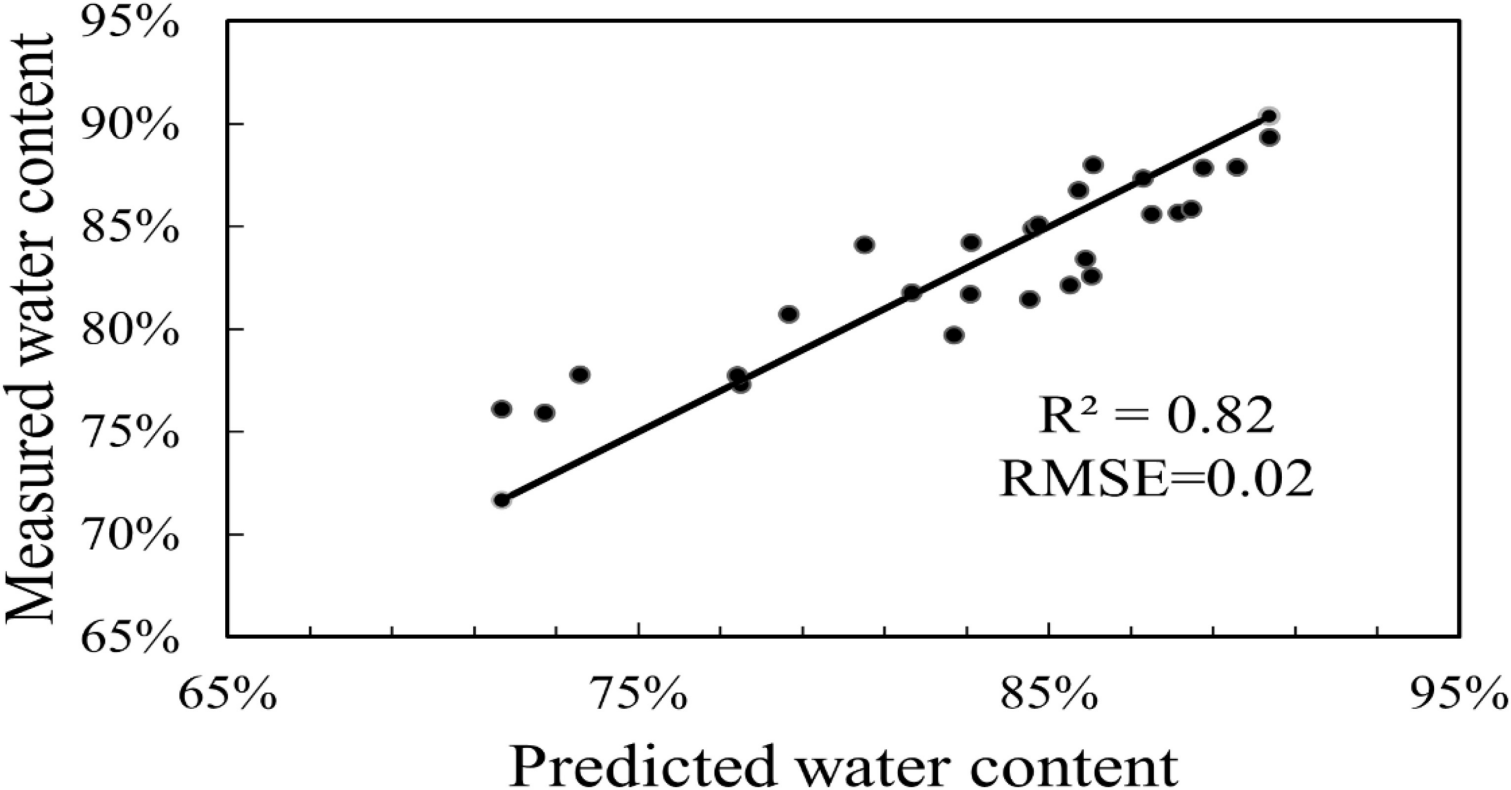
Verification of the quantitative relationship between T_2_ relaxation time and water content.

**FIGURE 6:**
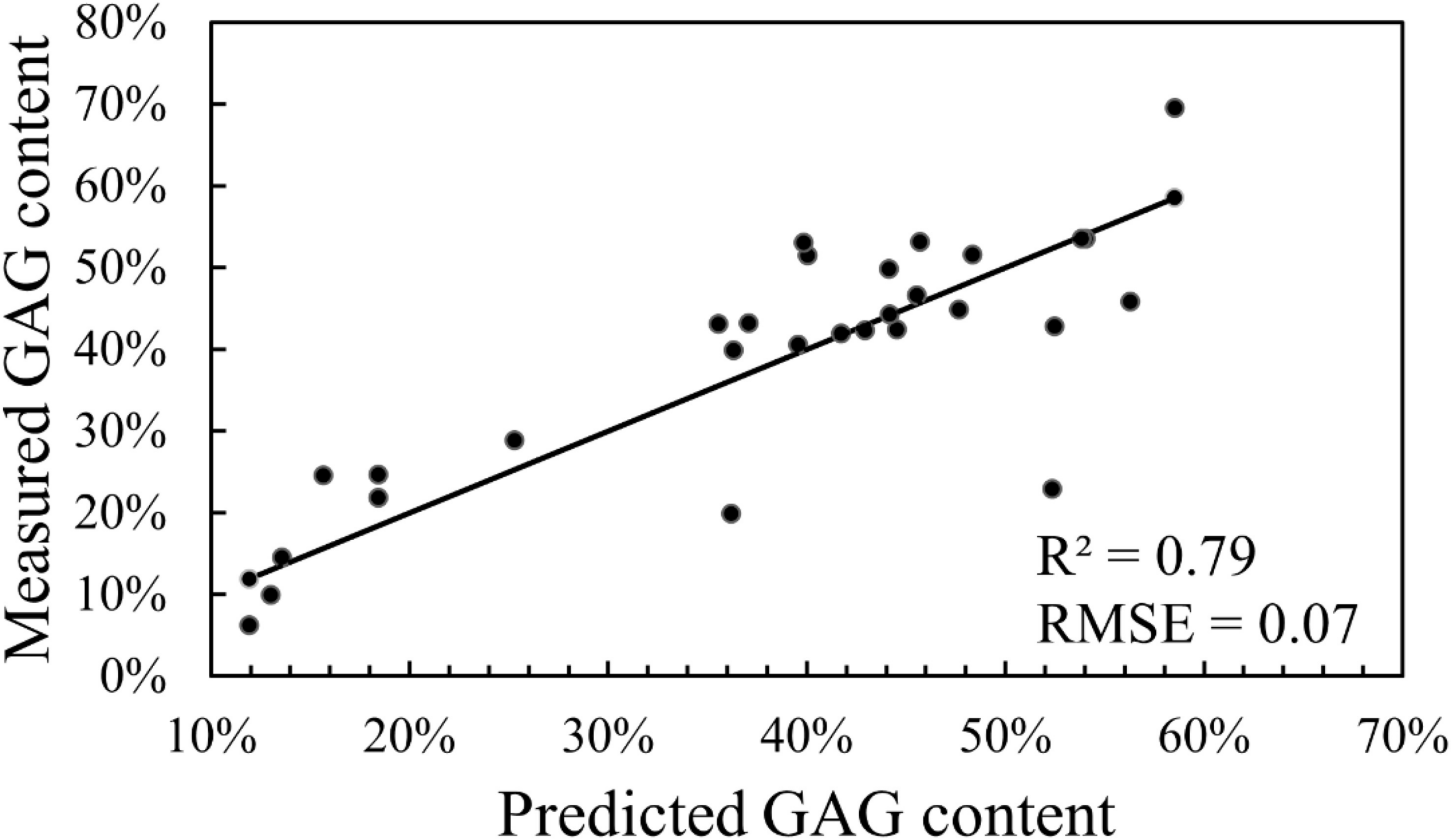
Verification of the quantitative relationship between T_2_ relaxation time and glycosaminoglycan (GAG) content.

The predictive accuracy of the linear regression models was assessed by comparing predicted and measured tissue composition values. For water content, the RMSE was 0.02 and R^2^ between predicted and measured values was 0.82 (Figure 5). For GAG content, the RMSE was 0.07 and R^2^ between predicted and measured values was 0.79 (Figure 6).

## Discussion

The water content and GAG content within the IVD were quantitatively analyzed using a high spatial resolution sampling approach based on matched T_2_ mapping and biochemical assays. We found that the T_2_ relaxation time, water content, and GAG content exhibited similar spatial distribution characteristics from the center to the outer IVD. We also found that T_2_ relaxation time showed statistically significant linear correlations with both water content and T_2_ relaxation time with GAG content. Furthermore, regression models derived from the correlations successfully predicted water and GAG content in an independent validation set. The present study integrated a 13-zone spatially matched sampling strategy with quantify evaluation of water and GAG contents based on the T_2_ relaxation time. This approach provided more spatially resolved MRI-biochemistry matching than previous T_2_ relaxation time -biochemistry correlation studies based on broad NP and AF regions.

The biochemical composition (water content and GAG content) and values of T_2_ relaxation time measured in the bovine caudal IVD in this study agreed well with previous studies on bovine IVD. Specifically, the water content (86% ± 4%), GAG content (44% ± 11%), and T_2_ relaxation time (92 ± 13 ms) were consistent with the ranges of 83% ± 4%, 35% ± 7% dry mass, and 83 ± 15 ms, respectively, reported for healthy bovine disks in the NP region, the water content (76% ± 4%), GAG content (24% ± 12%), and T_2_ relaxation time (60 ± 7 ms) were consistent with the ranges of 72% ± 2%, 13% ± 6% dry mass, and 43 ± 4 ms in the AF region (12, 13, 18). This quantitative consistency validates the reliability of our experimental and analytical methodology, thereby providing a basis for the subsequent correlation analyses and predictive modeling conducted in this study.

The observed spatial distributions of T_2_ relaxation time, water content, and GAG content were characterized by consistent radial gradients, with values highest in the central region and decreasing toward the peripheral AF. The spatial distribution pattern observed in this study corroborated the biochemical architecture of the IVD reported in the literature, where GAG content is highest in the central NP region and decreases toward the peripheral AF (9, 11). This is because the high concentration of negatively charged GAG generates a corresponding high fixed charge density and osmotic pressure within the IVD, drawing and retaining water, which maintained a high concentration of water content within the center of the IVD. High central water content was crucial for the IVD to bear and transfer loadings, and it is the basis of the biomechanical function of the IVD (5). In addition, the water within biological tissues was the principal determinant of the T_2_ relaxation time. This is because the T_2_ relaxation time is intrinsically governed by the mobility and local environment of water protons, both of which are directly dependent on hydration levels, regions of high free water content exhibit longer T_2_ relaxation time, a relationship demonstrated by various studies (12, 19). The application of a high spatial resolution sampling strategy in this study was critical for precisely capturing these continuous gradients of key biochemical components and T_2_ relaxation time.

The results showed significant associations between T_2_ relaxation time and both water and GAG content within the IVD. A strong linear correlation was observed between T_2_ relaxation time and water content (R^2^ = 0.8), which agreed with previous study (12), further validating T_2_ mapping as a reliable indicator for assessing water content within the IVD. A strong linear correlation was also observed between T_2_ relaxation time and GAG content (R^2^ = 0.74), which was slightly different from the conclusion of the weak association between T_2_ relaxation time and GAG content reported in previous studies (20), which may be due to the difference in sampling strategies. Compared to the conventional (NP/AF) dichotomy, our high spatial resolution sampling protocol could capture the inherent spatial heterogeneity of GAG distribution within the IVD (9, 21). This refined spatial characterization allows for a more accurate determination of GAG gradients, which significantly enhances the accuracy of quantitative assessments within the IVD. The strength of the T_2_ relaxation time -GAG correlation not only reflects their biochemical coupling via water mediation but also underscores the methodological advantage of spatially continuous sampling in elucidating the microscale matrix composition of the IVD. Importantly, T_2_ mapping does not directly measure GAG content. T_2_ relaxation time primarily reflects water proton mobility and the local extracellular matrix environment, whereas GAG may influence T_2_ relaxation time indirectly through its fixed charge density, associated hydration, and interactions with the extracellular matrix (3–5, 9, 12). Therefore, the observed T_2_ relaxation time -GAG association should be interpreted as an indirect, model-based relationship rather than a direct biochemical measurement of GAG. These findings provide a reference for advancing T_2_ relaxation time as a non-invasive biomarker capable of concurrently evaluating both the water content and GAG content within the IVD.

The predictive accuracy of the developed linear regression models was evaluated using an independent validation set. For water content, the RMSE was 0.02 and the R^2^ between predicted and measured values was 0.82. For GAG content, the RMSE was 0.07 with an R^2^ of 0.79. This predictive relationship enables the derivation of quantitative estimates for water and GAG contents directly from MRI, which is of significant value for IVD research. Specifically, this study provided a reference for quantitative research on biomechanical functions of the IVD, and also provided a non-invasive quantitative assessment method for biochemical components within IVD based on T_2_ mapping.

There were some limitations in this study. First, the quantitative relationships were explored and validated using *in vitro* bovine caudal IVD in this study. Although the bovine caudal IVD is similar to those of human, and have been widely studied, direct application of the specific quantitative relationships to *in vivo* human lumbar IVD requires further validation. Accordingly, this study provides a methodological reference for future research on human lumbar IVD, and future studies could establish spatially matched calibration relationships in a larger *ex vivo* human lumbar IVD cohort across degeneration grades, and subsequently assess their reproducibility and clinical relevance using *in vivo* T_2_ mapping with independent IVD-level validation. Second, while T_2_ mapping was sensitive to hydration, it is not a specific biomarker for GAG. Emerging techniques such as chemical exchange saturation transfer have shown more direct sensitivity to GAG. Future studies may improve accuracy by developing multiparametric MRI approaches for more precise, non-invasive quantification of both water and GAG content within the IVD. Finally, T_2_ relaxation time may be influenced by the orientation of collagen fibers relative to the main magnetic field (the magic-angle effect), B1-related refocusing imperfections, and water pools with distinct relaxation behavior within the IVD. These factors were not separately quantified or corrected in the present study and may have contributed to local variability in T_2_ relaxation time. Future studies could standardize specimen orientation relative to the main magnetic field, incorporate B1 mapping or refocusing-pulse correction, and use acquisition schemes suitable for multi-component relaxation analysis to clarify the respective contributions of tissue orientation, field inhomogeneity, and different water compartments.

In conclusion, a high spatial resolution sampling approach combined with T_2_ mapping and biochemical assays were applied to quantitatively analyze the correlations among T_2_ relaxation time, water content, and GAG content within the IVD in this study. The T_2_ relaxation time, water content, and GAG content showed similar distribution patterns across the IVD. A linear correlation was observed between T_2_ relaxation time and both water content and GAG content. These findings established a quantitative correlation between biochemical composition of the IVD with the intensity of the image signal, providing an *ex vivo* methodological foundation for the non-invasive quantification of both water and GAG contents within the IVD. This study contributed to the non-invasive quantification of IVD biomechanical function and image-based clinical diagnosis.

## Data Availability

The raw data supporting the conclusions of this article will be made available by the authors, without undue reservation.

## Footnotes

1
https://www.slicer.org
